# Efficiency of Therapeutic Plasma-Exchange in Acute Interstitial Lung Disease, Associated With Polymyositis/Dermatomyositis Resistant to Glucocorticoids and Immunosuppressive Drugs: A Retrospective Study

**DOI:** 10.3389/fmed.2019.00239

**Published:** 2019-11-05

**Authors:** Yaogui Ning, Guomei Yang, Yuechi Sun, Shiju Chen, Yuan Liu, Guixiu Shi

**Affiliations:** ^1^Department of Intensive Care Unit, The First Affiliated Hospital of Xiamen University, Xiamen, China; ^2^Medical College, Xiamen University, Xiamen, China; ^3^Department of Rheumatology and Clinical Immunology, The First Affiliated Hospital of Xiamen University, Xiamen, China

**Keywords:** therapeutic plasma exchange, interstitial lung disease, dermatomyositis, polymyositis, efficiency

## Abstract

Interstitial lung disease (ILD) is a life-threating complication, commonly associated with polymyositis (PM), and dermatomyositis (DM). A subset of acute ILD associated with PM/DM patients are refractory to conventional treatment, and leads to a high rate of mortality. The efficacy of therapeutic plasma-exchange (TPE) as a PM/DM treatment to improve muscle involvement is controversial due to a lack of evidence. However, in recent reports, TPE has been effective in improving lung involvement. To evaluate the efficacy of this therapy, we retrospectively studied TPE treatment outcomes for in 18 acute PM/DM-ILD patients who were resistant to conventional therapies. Five patients were diagnosed with DM (27.8%), 11 with CADM (61.1%), and two with PM (11.1%). Among 18 patients, 11 (61.1%) achieved satisfactory improvement after four or more rounds of TPE, whereas seven died due to respiratory failure. We also analyzed risk factors to predict unresponsiveness to TPE in these patients. Notably, the prevalence of subcutaneous/mediastinal emphysema was significantly higher in the non-responsive group (6/7, 85.7%) than in the responsive group (2/11, 18.2%; *P* = 0.013); moreover, patients with this complication were mainly in the CADM subgroup (6/8, 75%). Subcutaneous/mediastinal emphysema and increased serum ferritin levels were shown to be poor prognostic factors, predictive of unresponsiveness to TPE, in PM/DM patients. No autoantibodies were found to be associated with TPE outcome, although we only investigated anti-Jo-1 and anti-Ro antibodies; the clinical significance of other myositis-specific autoantibodies, especially anti-melanoma differentiation-associated gene 5 (MDA5) antibody, is not known. Our results indicate that TPE might be an alternative treatment for acute PM/DM-ILD patients resistant to conventional therapies, except for those with subcutaneous/mediastinal emphysema and high serum ferritin levels.

## Introduction

Idiopathic inflammatory myopathy (IIM) is a group of heterogeneous inflammatory muscle disorders, which includes subacute, chronic, and acute IIM. IIM is characterized by low muscle strength and endurance, as well as inflammatory cell infiltration into the skeletal muscles (1). Based on distinct clinical features, IIM can be subdivided into dermatomyositis (DM), polymyositis (PM), and inclusion body myositis (IBM). Clinically amyopathic dermatomyositis (CADM) is also defined in recent years and classified as a subgroup of DM. DM and PM are the most common forms of IIM and are also life-threating in rheumatic diseases. Besides the muscle and skin, other vital organs such as the lung and heart can also be involved in PM/DM, and complications in these organs contribute to the high rate of mortality associated with these conditions. Interstitial lung disease (ILD) is one of the most common and life-threating complications of PM/DM, with a prevalence up to 86% (2–6). The survival rate of patients with PM/DM-ILD is 56.7% during the first year, and even lower in those with acute ILD (7). Further, patients with rapidly progressive ILD are often resistant to high-dose glucocorticoids and immunosuppressive agents (8), thereby resulting in acute fatal respiratory failure with a 6-months survival rate of 40.8–45.0% (9, 10). The lack of effective treatment strategies for PM/DM-ILD patients who are resistant to glucocorticoids and immunosuppressive agents is the main contributor to high mortality rates.

Therapeutic plasma exchange (TPE) is a blood purification method that removes circulating cytokines, immune complexes, immunoglobulins, and complement components. Since the 1980s, it has been used to manage autoimmune diseases (11). However, the efficiency of TPE as a treatment for IIM remains controversial, due to a lack of evidence regarding its significant effects on improving muscle involvement (12–15). TPE is still not considered a standard treatment procedure for IIM. However, in recent years, it has shown promise. A patient with acute ILD associated with PM/DM was successfully treated and several case studies reported significant improvements in lung involvement after TPE treatment (16–20). Although evidence for TPE efficacy in PM/DM-ILD is limited, its potential as an effective treatment strategy for this disease is worth exploring, especially for patients who are resistant to glucocorticoids, and immunosuppressive agents. For a better understanding of the benefits of this treatment, we retrospectively studied the efficacy of this therapy based on 18 patients with acute PM/DM-ILD who were resistant to conventional therapies and specifically focused on the clinical characteristics of those who benefited from TPE.

## Methods

### Subjects

In this study, patients who were diagnosed with PM/DM-ILD resistant to conventional therapies and treated with TPE from January 2011 to May 2018 at the First Affiliated Hospital of Xiamen University were included. The conventional therapies included glucocorticoids and immunosuppressive agents. A total of 18 patients met the following inclusion criteria: (1) patients admitted into the intensive care unit (ICU) for ILD aggravation after failure of intensive treatment; (2) patients treated with TPE for more than four rounds. Patients with malignancy-associated disease, inclusion body myositis, and overlapping cases were excluded. The diagnosis of PM/DM was based on the Bohan and Peter diagnostic criteria, and diagnosis of clinically amyopathic dermatomyositis (CADM) was based on the diagnostic criteria of the European Neuromuscular Center international workshop (21), and the diagnosis of CADM, as a subtype of DM characterized by typical skin manifestations with little or no myositis, was based on the diagnostic criteria of the European Neuromuscular Center international workshop (22). CADM included both amyopathic dermatomyositis and hypomyopathic dermatomyositis. Amyopathic dermatomyositis is characterized by heliotrope rash, Gottron's papules, or Gottron's sign, and with normal creatine kinase (CK) and muscle biopsy results and no muscle weakness. Hypomyopathic dermatomyositis bears similar characteristic skin findings mentioned with no clinical evidence of muscle disease but mild changes in CK, magnetic resonance imaging (MRI), EMG, or muscle biopsy. Patients classified as having premyopathic dermatomyositis for whom fatal ILD developed within the first 6 months of their disease course were also included as CADM. All patients underwent muscle biopsies in the quadriceps. A chest high-resolution computed tomography (HRCT) was performed on each patient. ILD was diagnosed using HRCT imaging with the following qualitative criteria: signs of ground glass opacities, reticular abnormalities, traction bronchiectasis, irregular linear opacities, subpleural curvilinear shadows, and honeycombing. This retrospective study was approved by the Ethics Committee of the First Affiliated Hospital of Xiamen University, in accordance with the World Medical Association Declaration of Helsinki. Informed consent was obtained from either the patient or their authorized relative.

### Data Collection

The electronic medical record of each patient was retrospectively reviewed. The following data were collected: information of patients' characteristics, such as age, sex, and disease course of PM/DM; clinical symptoms including IIM-related and pulmonary symptoms; occurrence of subcutaneous/mediastinal emphysema; Acute Physiology and Chronic Health Evaluation (APACHE) II score (23) during the first 24 h after admission to the ICU; type of immunosuppressive therapy administered; use of ventilator assistance; laboratory findings.

### TPE Procedures

TPE procedures were performed every day for 3 days, and every other day after that, using the AQUARIUS multifiltrate machine (Edwards Lifesciences AG, Irvine, CA, USA). Intravenous methylprednisolone (40–80 mg/days) and oral immunosuppressive agents were administered as a combined maintenance therapy. For vascular access, a double coaxial lumen 14-Fr catheter was inserted percutaneously, either through the right or left femoral vein, using the Seldinger technique. The blood flow rate was 80 mL/min for TPE. Plasma (40–60 mL plasma/kg) was exchanged for the same volume of normal fresh frozen plasma each time. The duration of TPE was 3–4 h. The procedures were performed by trained nurses and supervised by senior physicians at the ICU. Treatment was suspended when a significant improvement in CT or death occurred.

### Statistical Analysis

All statistical calculations were conducted using SPSS 23.0 software. Data are presented as the mean ± standard error of mean (SEM), or median (range) for continuous variables, and numbers (percentages) for qualitative variables. For comparisons between two groups, the chi-squared, or Fisher's exact tests were used for binary data and the Student's *t*- or Mann-Whitney *U*-tests were used for continuous data. Results of the logistic regression models are shown as the odds ratio (OR) and 95% confidence interval (CI). A *p* < 0.05 indicated statistical significance.

## Results

### Efficacy of TPE for Acute PM/DM-ILD Patients Resistant to Conventional Therapies

**Figure 1:**
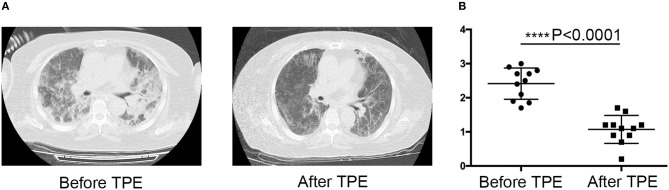
Effect of therapeutic plasma exchange (TPE) on polymyositis (PM) and dermatomyositis interstitial lung disease (PM/DM-ILD) improvement. **(A)** Representative CT images of the lung before and after TPE. Lung CT scans of one patient before and after TPE. Interstitial opacities with multifocal ground glass opacities and consolidations (left panel). Follow-up CT scan indicating the frank regression of interstitial pneumonia (right panel). **(B)** CT score before and after TPE treatment in the responsive group (*n* = 11), ****p < 0.0001.

This retrospective study included 18 patients who received TPE for the aggravation of ILD after treatment with a combination of high-dose glucocorticoids, cyclophosphamide, a calcineurin inhibitor, or intravenous immunoglobulin G. Five patients were diagnosed with DM (27.8%), 11 with CADM (61.1%), and two with PM (11.1%). The main respiratory symptom was dyspnea on exertion. Fine crackles were also observed in these patients. Although seven patients (38.9%) died from respiratory failure after TPE, the other 11 patients (61.1%) showed great improvement in lung involvement, reduced HRCT scores (24, 25), and their conditions were not life-threatening after treatment (Figure 1). These data suggested that TPE might be an alternative treatment strategy for acute PM/DM-ILD patients resistant to conventional therapies.

### Clinical Characteristics of PM/DM-ILD Patients Responsive to TPE

We analyzed the characteristics and clinical profiles of the PM/DM-ILD patients whose conditions were improved by TPE. We divided PM/DM-ILD patients into responsive (*n* = 11) and non-responsive (*n* = 7) groups. Responsiveness was defined as improved or controlled lung involvement and rescue from life-threating complications, whereas non-responsiveness was defined as aggressive lung involvement and death. The clinical characteristics of the patients are summarized in Table 1.

**Table 1:** Comparison of clinical characteristics between PM/DM-ILD patients who were responsive and non-responsive to TPE.

**Variables**	**Responsive group (*n* = 11)**	**Non-responsive group (*n* = 7)**	***P*-value**
Sex, male/female, *n* (%)	3/8 (27.3/72.7)	3/4 (42.9/57.1)	0.627
Age, years, mean ± SEM	55.70 ± 11.08	52.71 ± 11.46	0.540
**DISEASE DURATION, WEEKS, MEDIAN (RANGE)**			
at ILD diagnosis	3.0 (1–4)	3.2 (1.57–5.71)	0.328
at PM/DM/CADM diagnosis	13 (2.43–96)	6.86 (4–528)	0.536
**IIM TYPE**, ***n*** **(%)**			
PM/DM	2/3 (18.2/27.3)	0/2 (0/28.6)	0.952
CADM	6 (54.5)	5 (71.4)	0.637
**CLINICAL SYMPTOM**, ***n*** **(%)**			
Arthritis/arthralgia	4 (36.4)	1 (14.3)	0.596
Skin rash	9 (81.8)	6 (85.7)	1.000
Fever	3 (27.3)	2 (28.6)	1.000
Cough	4 (36.4)	3 (42.9)	1.000
Dyspnea on exertion	9 (81.8)	6 (85.7)	1.000
Dysphagia	3 (27.3)	1 (14.3)	1.000
Muscle weakness/myalgia	5 (45.5)	1 (14.3)	0.316
Subcutaneous/mediastinal emphysema *n* (%)	2 (18.2)^*^	6 (85.7)	0.013
APACHE II Score, median (range)	17 (11–24)	18.5 (15–31)	0.126
P/F ratio	218.8 ± 13.38	173.3 ± 21.38	0.074
**THERAPY**, ***n*** **(%)**			
High-dose steroids	11 (100)	7 (100)	NA
Cyclosporine A	8 (72.7)	5 (71.4)	1.000
Cyclophosphamide	6 (54.5)	2 (28.6)	0.367
Intravenous immunoglobulin G	6 (54.5)	5 (71.4)	0.637
Hydroxychloroquine	1 (9.1)	1 (14.3)	1.000
Methotrexate	1 (9.1)	0	0.611
Thalidomide	2 (18.2)	0	0.137
Total dosage of MP before TPE, mg (mean ± SEM)	460.9 ± 49.88	341.4 ± 61.81	0.153
Duration of MP use before TPE, days (mean ± SEM)	6.6 ± 0.3	6.4 ± 0.4	0.676
**PLASMA EXCHANGE**			
Times, median (range)	5 (4–24)	6 (4–10)	0.724
Plasma amount, mL, median (range)	3,000 (2,500–3,000)	3,000 (2,500–3,000)	0.724
Use of ventilator, *n* (%)	4 (36.4)	6 (85.7)	0.066

*ILD, Interstitial lung disease; PM, polymyositis; DM, dermatomyositis; CADM, clinically amyopathic dermatomyositis; IIM, idiopathic inflammatory myopathy; SEM, standard error of mean; TPE, therapeutic plasma-exchange; APACHE II, Acute Physiology and Chronic Health Evaluation; MP, methylprednisolone; P/F, arterial partial pressure of oxygen /fraction of inspired oxygen*.

* *p < 0.05*.

No significant differences were observed between the responsive and non-responsive groups in terms of other clinical parameters such as age, types of IIM, and disease duration (Table 1). In the two groups, the most common IIM type was CADM (54.5 and 71.4%, respectively). Skin lesions were observed in nine cases in the responsive group (81.8%) and six in the non-responsive group (85.7%), including skin ulceration (three and three, respectively), palmar papules (four and three), oral erosions (one and zero), heliotrope rash (four and two), and Gottron papules (six and five). Skin ulceration, palmar papules, and oral erosions are unique cutaneous phenotypes associated with the anti-melanoma differentiation associated protein 5 (MDA5) antibody (26); regarding these rashes, there were no significant differences between the two groups.

Notably, six patients (five CADM and one DM) of seven patients in the non-responsive group suffered from mediastinal emphysema; only two (one CADM and one DM) of 11 patients in the responsive-group had this complication. Patients with SP were mainly in the CADM subgroup (6/8, 75%). Three cases in the non-responsive group had SP concomitant subcutaneous emphysema. The prevalence of subcutaneous/mediastinal emphysema was significantly higher in the non-responsive group (85.7%) than in the responsive group (18.2%), suggesting that subcutaneous/mediastinal emphysema might be a treatment response predictor for TPE.

### Laboratory Characteristics of PM/DM-ILD Patients Responsive to TPE

Laboratory findings of PM/DM-ILD patients receiving TPE are shown in Table 2. Antinuclear antibodies (≥1: 100) were detected in three patients (16.7%). The myositis-specific autoantibody, anti-Jo-1, was present in only one patient (5.6%). Regarding myositis-associated autoantibodies, anti-SSA/Ro antibodies were identified in 12 patients (66.7%). In four patients (22.2%), no antibodies were detected. The CD4+/CD8+ T ratio was significantly higher in the responsive group than in the non-responsive group (*p* = 0.049), implying that TPE might have exerted little effects on PM/DM-ILD patients whose pathogeneses were mainly attributed to CD8+ T cells. Levels of C-reactive protein and serum ferritin were significantly lower in the responsive group than in the non-responsive group (*p* = 0.031 and *p* = 0.002, respectively). Besides the three mentioned parameters, no other significant differences between the groups were identified.

**Table 2:** Comparison of laboratory characteristics between responsive and non-responsive groups of PM/DM-ILD patients.

**Clinical parameters**	**Responsive group (*n* = 11)**	**Non-responsive group (*n* = 7)**	**P-value**
Lymphocytes, × 10^9^/L, median (range)	0.69 (0.38–9.50)	0.60 (0.12–1.16)	0.285
CD4+/8+ T ratio, mean ± SEM	2.01 ± 0.58^*^	1.29 ± 0.87	0.049
Platelet count, × 10^9^/L, mean ± SEM	224.819 ± 85.427	203.571 ± 91.874	0.894
Erythrocyte sedimentation rate, mm/h, median (range)	29 (2–105)	30 (1–64)	0.660
C-reactive protein, mg/L, mean ± SEM	6.506 ± 5.056^*^	15.281 ± 8.170	0.031
Serum ferritin, μg/L, median (range)	414.6 (78.1–3659.4)^*^	1518.6 (984.2–3819.2)	0.002
IL-6, pg/mL, median (range)	3.58 (0.07–35.50)	19.77 (5.99–832)	0.247
Procalcitonin (PCT), ng/mL, median (range)	0.710 (0.037–0.655)	0.125 (0.036–7.520)	0.151
Serum albumin (ALB), mg/L, mean ± SEM	31.960 ± 3.289	32.486 ± 3.023	0.204
Alanine aminotransferase (ALT), IU/L, median (range)	99 (20–142)	60 (26–439)	0.659
Aspartate aminotransferase (AST), IU/L, median (range)	64 (20–100.5)	60 (24–467)	0.860
Creatine kinase, IU/L, median (range)	80 (10–3,794)	83 (46–770)	0.930
Lactate dehydrogenase (LDH), IU/L, median (range)	412 (58–1,337)	491 (312–2,032)	0.375
Creatine, IU/L, mean ± SEM	54.82 ± 21.10	107.14 ± 128.4	0.325
Positive antinuclear antibody, *n* (%)	3(27.3)	0	0.245
Positive anti-Jo-1 antibody, *n* (%)	1(9.1)	0	0.611
Anti-SSA antibody, positivity, *n* (%)	7 (63.5)	5 (71.4)	1.000
Anti Ro-52 antibody, *n* (%)	7 (63.5)	4 (57.1)	1.000
Immunoglobulin A, mg/dL, median (range)	1.78 (1.39–3.55)	1.91 (0.72–3.65)	1.000
Immunoglobulin M, mg/dL, median (range)	1.45 (0.765–2.05)	1.100 (0.245–8.900)	0.425
Immunoglobulin G, mg/dL, mean ± SEM	14.84 ± 5.97	8.75 ± 6.15	0.894

* *p < 0.05. SEM, standard error of mean; ILD, interstitial lung disease; PM, polymyositis; DM, dermatomyositis*.

### HRCT Findings in PM/DM-ILD Patients Responsive to TPE

HRCT imaging characteristics of all patients are shown in Table 3. Ground glass opacities, irregular linear opacities, and consolidation were the main image findings in these patients. No significant differences in HRCT features of PM/DM-ILD were observed between the responsive and non-responsive groups. The condition of most patients was too serious for them to undergo pulmonary function tests. Figure 1 shows improvements in the CT scores of survivors before and after TPE treatment (2.414 ± 0.1379 and 1.073 ± 0.1236, respectively, *p* < 0.0001).

**Table 3:** Comparison of HRCT findings between responsive and non-responsive groups of PM/DM-ILD patients.

**CT findings**	**Responsive group (*n* = 11)**	**Non-responsive group (*n* = 7)**	***P*-value**
Consolidation, *n* (%)	9 (81.8)	6 (85.7)	1.000
Ground glass opacities, *n* (%)	5 (45.5)	5 (71.4)	0.367
Irregular linear opacities, *n* (%)	8 (72.7)	5 (71.4)	1.000
Traction bronchiectasis, *n* (%)	0	2 (28.6)	0.137
Honeycombing, *n* (%)	1 (9.1)	1 (14.3)	1.000
Subpleural curvilinear shadows, *n* (%)	0	1 (14.3)	0.389

*ILD, interstitial lung disease; PM, polymyositis; DM, dermatomyositis; HRCT, high-resolution computed tomography*.

### Risk Factors to Predict TPE Efficiency

We next evaluated the risk factors that could predict the unresponsiveness of PM/DM-ILD patients to TPE treatment. The results of univariate analysis revealed that four parameters, namely subcutaneous/mediastinal emphysema, CD4+/8+ ratio, and CRP and serum ferritin levels, were significantly different between the responsive and non-responsive groups. A multivariable logistic model was then established to predict the risk factors related to patient unresponsiveness to TPE (Table 4). The results of logistic regression analyses showed that subcutaneous/mediastinal emphysema and serum ferritin levels were significantly associated with this in acute PM/DM-ILD patients who were resistant to conventional therapies. CRP levels and the CD4+/8+ ratio were found to be risk factors for death.

**Table 4:** Adjusted odds ratios (ORs) with associated 95% confidence interval (95%CI) for death.

**Variables**	**Death**		
	**OR**	**95%CI**	***P*-value**
CD4+/8+ T cell ratio	0.188	0.030–1.164	0.072
C-reactive protein (mg/L)	1.351	0.972–1.878	0.073
Subcutaneous/mediastinal emphysema	15.185	1.233–186.983	0.034^*^
Serum ferritin (μg/L)*^†^*	5.683	1.110–29.101	0.037^*^

* *p < 0.05. Model was adjusted for sex and age*.

† *OR and 95% CI are expressed by standard deviation increases in serum ferritin*.

## Discussion

ILD is very common in PM/DM patients and can cause life-threatening complications even after standard treatments. A large proportion of patients with acute PM/DM-ILD show no response to conventional therapies including glucocorticoids and immunosuppressive agents, leading to uncontrolled and aggressive lung involvement and finally death due to respiratory failure. This is the first and largest retrospective study to analyze the efficacy of TPE therapy for acute PM/DM-ILD patients who were resistant to conventional therapies and to evaluate the risk factors that can predict unresponsiveness to this treatment. Our study showed that TPE might be an alternative treatment for acute PM/DM-ILD patients who are resistant to conventional therapies.

TPE was initially developed to treat liver failure and immune diseases. The use of TPE against PM/DM has been controversial for years. The American Society for Apheresis' indication category for TPE use in PM/DM was IV in the latest 2016 Therapeutic Apheresis guideline (27). To date, this recommendation is based on the results of a unique randomized controlled trial comprising 39 PM/DM patients by Miller et al. (12). In that study, there was no significant difference in final muscle strength or functional capacity following plasma exchange, leukapheresis, or sham apheresis. No concomitant immunosuppressants except glucocorticoids were administered to all patients. In 1981, Dau, in an uncontrolled trial, treated 35 inflammatory myopathy patients with TPE combined with immunosuppressants (cyclophosphamide or chlorambucil), and found improvement in muscle strength without significant side effects in 32 of them (13). Other retrospective multicenter studies have also demonstrated the efficiency of TPE in PM/DM. Herson examined 38 PM/DM patients who were treated with TPE as a rescue therapy when conventional treatment failed, and observed improvements in muscle strength in 24 (63%) patients (14). Cherin investigated 27 patients who suffered from severe pharyngeal muscle weakness and were resistant to conventional therapy; eight (30%) reported the disappearance of symptoms, whereas the other 19 (70%) reported the stabilization of dysphagia after receiving TPE (15). Some case reports have also showed that TPE in association with immunosuppressant agents could play a relevant role in severe pharyngo-esophageal muscle weakness (28).

The effects of TPE on acute respiratory failure during ILD have not been fully established. In 2015, Omotoso published a report in which TPE was found to be beneficial for the treatment of a patient with ILD-associated antisynthetase syndrome who was refractory to glucocorticoids and other immunosuppressive therapeutics (17). Bozkirli also reported a case of antisynthetase syndrome with ILD who benefited from double-filtration plasmapheresis (16). The therapeutic effects of TPE also include the removal of pathological substances from the blood, such as autoantibodies, cytokines, complement components, and paraproteins. Further, other possible mechanisms include alterations to lymphocyte proliferation, the immune system, and cell sensitivity to immunosuppressants or chemotherapeutic agents (29–31). Although more data are necessary, TPE might be an immediate treatment option for acute PM/DM-ILD patients who are resistant to conventional therapies. Moreover, because TPE substitutes fresh frozen plasma components such as anti-idiotypic antibodies and immunoglobulins, which target host antigens, this therapy might provide additional therapeutic benefits (29). Clearly, TPE is only a short-term solution, because immune cells that secrete antibodies, complement components, and cytokines will continue to function in response to repeated antigenic stimulation after TPE. Moreover, the transient effects of TPE require additional long-term immunosuppression treatment. Another disadvantage of this therapy is risks associated with the use of blood products, including sexually transmitted diseases. Despite these drawbacks, no plasma-related adverse events were observed in patients after short-term treatment in the current study.

In the present study, a multivariable logistic model showed that subcutaneous/mediastinal emphysema and serum ferritin levels were significantly associated with unresponsiveness to TPE. A previous study showed that serum ferritin level is the most significant prognostic factor for PM/DM (32). Moreover, serum ferritin was found to predict the disease severity and prognosis for anti-MDA5 antibody-associated ILD with DM; a serum ferritin concentration cut-off value of 1,600 μg/L was suggested to be the best indicator of survival in this subgroup (33). In the present study, the average ferritin level in the unresponsiveness group was 1518.6 μg/L, almost equal to that value. However, the lack of an anti-MDA5 antibody test did not allow us to conclude whether patients in the unresponsiveness group had anti-MDA5 antibody-associated ILD. In addition, PM/DM-ILD patients with hyperferritinemia might be unresponsive to TPE when resistant to conventional therapies.

Subcutaneous/mediastinal emphysema was found to be another potential prognostic factor associated with TPE outcome in our study. DM/PM patients are mostly predisposed to develop spontaneous pneumomediastinum with a prevalence ranging from 2.2 to 8.6% (10, 34–36). Spontaneous pneumomediastinum can be fatal if unrecognized and can lead to death within 2 months in approximately 25% of patients (34, 35); further, it is more prevalent in patients with CADM (34). In this retrospective study, six patients with spontaneous pneumomediastinum (6/8, 75%) were diagnosed with CADM. CADM patients should be carefully screened for spontaneous pneumomediastinum since the latter is a prognostic factor. A previous study also demonstrated that anti-MDA5 antibodies are associated with spontaneous pneumomediastinum (35). Spontaneous pneumomediastinum increases the risk of death in DM patients with anti-MDA5 antibody-associated ILD (37). These findings again indicate that anti-MDA5 antibodies might be associated with TPE outcome. However, anti-MDA5 and other myositis-specific autoantibodies were not investigated in the present study. In the responsive group, two patients suffered from spontaneous pneumomediastinum and one patient died due to respiratory failure several months after discharge from the hospital. Taken together, these results suggest that spontaneous pneumomediastinum is a poor prognostic factor for TPE and that patients who suffer from this could comprise a population that should be excluded from TPE treatment.

There are some limitations to the present retrospective study. The high cost of TPE imposed restrictions on its application, thus limiting the size of our patient sample size. Further, the lack of data on most myositis-specific antibodies, and especially anti-MDA5 antibody testing, in these patients did not allow us to conclude whether the anti-MDA5 antibody was a predictive factor of TPE outcome. It was reported that forced vital capacity is a poor predictive factor for survival with ILD (38). In this retrospective study, pulmonary function tests including carbon monoxide-diffusing capacity were unavailable due to the patients' severe disease states. Moreover, measurements of Krebs von den Lungen-6 levels were not performed; therefore, the severity of ILD could not be assessed. Those limitations resulted in these important parameters being excluded from predictive evaluation.

In conclusion, this retrospective study shows promise regarding the use of TPE in addition to glucocorticoids and immunosuppressants for early-stage PM/DM-ILD. Further, subcutaneous/mediastinal emphysema and serum ferritin levels might serve as poor prognostic factors of responsiveness to TPE. More controlled trials and long-term observations are required in the future.

## Data Availability Statement

The raw data supporting the conclusions of this manuscript will be made available by the authors, without undue reservation, to any qualified researcher.

## Ethics Statement

This study was carried out in accordance with the recommendations of the Ethics Committee of the First Affiliated Hospital of Xiamen University with written informed consent from all subjects. All subjects gave written informed consent in accordance with the Declaration of Helsinki.

## Author Contributions

YN, SC, and GS conceived and designed this study. YN and YL were responsible for the integrity of the study, interpretation of data, and drafting of the manuscript. YN, GY, and YS participated in medical record collection. All authors reviewed and approved the manuscript for submission.

### Conflict of Interest

The authors declare that the research was conducted in the absence of any commercial or financial relationships that could be construed as a potential conflict of interest.

## References

[1] Plotz PH Dalakas M Leff RL Love LA Miller FW Cronin ME. Current concepts in the idiopathic inflammatory myopathies: polymyositis, dermatomyositis, and related disorders. Ann Intern Med. (1989) 111:143–57. 2662848 10.7326/0003-4819-111-2-143

[2] Fathi M Lundberg IE. Interstitial lung disease in polymyositis and dermatomyositis. Curr Opin Rheumatol. (2005) 17:701–6. 10.1097/01.bor.0000179949.65895.53 16224246

[3] Fathi M Lundberg IE Tornling G. Pulmonary complications of polymyositis and dermatomyositis. Semin Respir Crit Care Med. (2007) 28:451–8. 10.1055/s-2007-985666 17764062

[4] Morisset J Johnson C Rich E Collard HR Lee JS. Management of myositis-related interstitial lung disease. Chest. (2016) 150:1118–28. 10.1016/j.chest.2016.04.007 27102182

[5] Marie I Lahaxe L Benveniste O Delavigne K Adoue D Mouthon L. Long-term outcome of patients with polymyositis/ dermatomyositis and anti-PM-Scl antibody. Br J Dermatol. (2010) 162:337–44. 10.1111/j.1365-2133.2009.09484.x 19845665

[6] Douglas WW Tazelaar HD Hartman TE Hartman RP Decker PA Schroeder DR. Polymyositis-dermatomyositis-associated interstitial lung disease. Am J Respir Crit Care Med. (2001) 164:1182–5. 10.1164/ajrccm.164.7.2103110 11673206

[7] Chen IJ Jan Wu YJ Lin CW Fan KW Luo SF Ho HH. Interstitial lung disease in polymyositis and dermatomyositis. Clin Rheumatol. (2009) 28:639–46. 10.1007/s10067-009-1110-6 19247576

[8] Schnabel A Reuter M Biederer J Richter C Gross WL. Interstitial lung disease in polymyositis and dermatomyositis: clinical course and response to treatment. Semin Arthritis Rheum. (2003) 32:273–84. 10.1053/sarh.2002.50012 12701038

[9] Mukae H Ishimoto H Sakamoto N Hara S Kakugawa T Nakayama S. Clinical differences between interstitial lung disease associated with clinically amyopathic dermatomyositis and classic dermatomyositis. Chest. (2009) 136:1341–7. 10.1378/chest.08-2740 19581351

[10] Ye S Chen XX Lu XY Wu MF Deng Y Huang WQ. Adult clinically amyopathic dermatomyositis with rapid progressive interstitial lung disease: a retrospective cohort study. Clin Rheumatol. (2007) 26:1647–54. 10.1007/s10067-007-0562-9 17308858

[11] Dequeker J Geusens P Wielands L. Short and longterm experience with plasmapheresis in connective tissue diseases. Biomedicine. (1980) 32:189–94. 7470581

[12] Miller FW Leitman SF Cronin ME Hicks JE Leff RL Wesley R. Controlled trial of plasma exchange and leukapheresis in polymyositis and dermatomyositis. N Engl J Med. (1992) 326:1380–4. 10.1056/NEJM199205213262102 1472183

[13] Dau PC. Plasmapheresis in idiopathic inflammatory myopathy: experience with 35 patients. JAMA Neurol. (1981) 38:544–52. 10.1001/archneur.1981.00510090038003 7271533

[14] Herson S Lok C Roujeau JC Coutellier A Etienne SD Revuz J. Plasma exchange in dermatomyositis and polymyositis. Retrospective study of 38 cases of plasma exchange. Ann Med Interne. (1989) 140:453–5. 2696399

[15] Cherin P Auperin I Bussel A Pourrat J Herson S. Plasma exchange in polymyositis and dermatomyositis: a multicenter study of 57 cases. Clin Exp Rheumatol. (1995) 13:270–1. 7656479

[16] Bozkirli DEE Kozanoglu I Bozkirli E Yucel E. Antisynthetase syndrome with refractory lung involvement and myositis successfully treated with double filtration plasmapheresis. J Clin Apher. (2013) 28:422–5. 10.1002/jca.21285 23908096

[17] Omotoso BA Ogden MI Balogun RA. Therapeutic plasma exchange in antisynthetase syndrome with severe interstitial lung disease. J Clin Apher. (2015) 30:375–9. 25727180 10.1002/jca.21387

[18] Fujita Y Fukui S Suzuki T Ishida M Endo Y Tsuji S. Anti-MDA5 antibody-positive dermatomyositis complicated by autoimmune-associated hemophagocytic syndrome that was successfully treated with immunosuppressive therapy and plasmapheresis. Intern Med. (2018) 57:3473–78. 10.2169/internalmedicine.1121-18 29984753 PMC6306524

[19] Endo Y Koga T Suzuki T Hara K Ishida M Fujita Y. Successful treatment of plasma exchange for rapidly progressive interstitial lung disease with anti-MDA5 antibody-positive dermatomyositis: a case report. Medicine. (2018) 97:e0436. 10.1097/MD.0000000000010436 29642214 PMC5908626

[20] Yagishita M Kondo Y Terasaki T Terasaki M Shimizu M Honda F. Clinically amyopathic dermatomyositis with interstitial pneumonia that was successfully treated with plasma exchange. Intern Med. (2018) 57:1935–8. 10.2169/internalmedicine.0297-17 29491297 PMC6064687

[21] Bohan A Peter JB Bohan A Peter JB Polymyositis and dermatomyositis. Parts 1 and 2. N Engl J Med. (1975) 292:344–7. 10.1056/NEJM197502132920706 1090839

[22] Bailey EE Fiorentino DF. Amyopathic dermatomyositis: definitions, diagnosis, and management. Curr Rheumatol Rep. (2014) 16:1–7. 10.1007/s11926-014-0465-0 25366932

[23] Knaus WA Draper EA Wagner DP Zimmerman JE. APACHE II: a severity of disease classification system. Crit Care Med. (1985) 13:818–29. 10.1097/00003246-198510000-00009 3928249

[24] Ichikado K Suga M Muranaka H Gushima Y Miyakawa H Tsubamoto M. Prediction of prognosis for acute respiratory distress syndrome with thin-section CT: validation in 44 cases. Radiology. (2006) 238:321–9. 10.1148/radiol.2373041515 16293804

[25] Ichikado K Suga M Muller NL Taniguchi H Kondoh Y Akira M Acute interstitial pneumonia: comparison of high-resolution computed tomography findings between survivors and non-survivors. Am J Respir Crit Care Med. (2002) 165:1551–6. 10.1164/rccm.2106157 12045132

[26] Muro Y Sugiura K Akiyama M. Cutaneous manifestations in dermatomyositis: Key clinical and serological features-a comprehensive review. Clin Rev Allergy Immunol. (2016) 51:293–302. 10.1007/s12016-015-8496-5 26100618

[27] Schwartz J Padmanabhan A Aqui N Balogun RA Connelly-Smith L Delaney M. Guidelines on the use of therapeutic apheresis in clinical practice—evidence-based approach from the Writing Committee of the American society for apheresis: the seventh special issue. J Clin Apher. (2016) 31:149–62. 10.1002/jca.21470 27322218

[28] Cozzi F Marson P Pigatto E Tison T Polito P Galozzi P. Plasma-exchange as a rescue therapy for dermato/polymyositis in acute phase. Experience in three young patients. Transfus Apher Sci. (2015) 53:368–72. 10.1016/j.transci.2015.07.005 26283176

[29] Reeves HM Winters JL. The mechanisms of action of plasma exchange. Br J Haematol. (2014) 164:342–51. 10.1111/bjh.12629 24172059

[30] Nakamura T Ushiyama C Suzuki S Shimada N Ebihara I Suzaki M. Effect of plasma exchange on serum tissue inhibitor of metalloproteinase 1 and cytokine concentrations in patients with fulminant hepatitis. Blood Purif. (2000) 18:50–4. 10.1159/000014407 10686442

[31] Toft P Schmidt R Broechner AC Nielsen BU Bollen P Olsen KE. Effect of plasmapheresis on the immune system in endotoxin-induced sepsis. Blood Purif. (2008) 26:145–50. 10.1159/000113507 18212497

[32] Ishizuka M Watanabe R Ishii T Machiyama T Akita K Fujita Y. Long-term follow-up of 124 patients with polymyositis and dermatomyositis: statistical analysis of prognostic factors. Mod Rheumatol. (2016) 26:115–20. 10.3109/14397595.2015.1054081 26011440

[33] Gono T Kawaguchi Y Satoh T Kuwana M Katsumata Y Takagi K. Clinical manifestation and prognostic factor in anti-melanoma differentiation-associated gene 5 antibody-associated interstitial lung disease as a complication of dermatomyositis. Rheumatology. (2010) 49:1713–9. 10.1093/rheumatology/keq149 20498012

[34] Le Goff B Cherin P Cantagrel A Gayraud M Hachulla E Laborde F. Pneumomediastinum in interstitial lung disease associated with dermatomyositis and polymyositis. Arthritis Rheum. (2009) 61:108–18. 10.1002/art.24372 19116970

[35] Ma X Chen Z Hu W Guo Z Wang Y Kuwana M. Clinical and serological features of patients with dermatomyositis complicated by spontaneous pneumomediastinum. Clin Rheumatol. (2016) 35:489–93. 10.1007/s10067-015-3001-3 26149923

[36] Kono H Inokuma S Nakayama H Suzuki M. Pneumomediastinum in dermatomyositis: association with cutaneous vasculopathy. Ann Rheum Dis. (2000) 59:372–6. 10.1136/ard.59.5.372 10784520 PMC1753137

[37] Yamaguchi K Yamaguchi A Itai M Kashiwagi C Takehara K Aoki S. Clinical features of patients with anti-melanoma differentiation-associated gene-5 antibody-positive dermatomyositis complicated by spontaneous pneumomediastinum. Clin Rheumatol. (2019). 10.1007/s10067-019-04729-5. [Epub ahead of print]. 31420814

[38] Kang EH Lee EB Shin KC Im CH Chung DH Han SK. Interstitial lung disease in patients with polymyositis, dermatomyositis and amyopathic dermatomyositis. Rheumatology. (2005) 44:1282–6. 10.1093/rheumatology/keh723 15972351

